# Isolated Pericardial Effusion Without Associated Myocarditis in a Small-Cell Lung Cancer Patient Undergoing Atezolizumab Therapy

**DOI:** 10.7759/cureus.60184

**Published:** 2024-05-13

**Authors:** Kiara Jamison, Lalitha C Medepalli, Star Ye

**Affiliations:** 1 Internal Medicine, Northside Hospital Gwinnett, Lawrenceville, USA; 2 Cardiology/Cardiooncology, Northside Cardiovascular Institute (NCVI) Northside Hospital, Atlanta, USA; 3 Oncology, Georgia Cancer Specialists (Affiliated With Northside Hospital Cancer Institute), Canton, USA

**Keywords:** small cell lung cancer (sclc), pericardial effusion, immune-related adverse events, programmed death, immune checkpoint inhibitors

## Abstract

Immune checkpoint inhibitors (ICIs) are a form of immunotherapy increasingly utilized in cancer therapies. While offering promise in malignancy treatment, ICIs, including atezolizumab, can elicit immune-related adverse events (irAEs) such as cardiotoxicity. We present the case of a 67-year-old male with stage IV metastatic small-cell lung cancer undergoing carboplatin, etoposide, and atezolizumab therapy, who developed pericardial tamponade two months into treatment. Initially presenting with hypoxia on day three of his third treatment cycle, he was admitted due to multifocal pneumonia and subsequently diagnosed with pericardial tamponade stemming from a sizable pericardial effusion. Pericardiocentesis was performed, effectively resolving the tamponade. Infectious etiology was ruled out. Notably, there was no associated myocarditis, as evidenced by negative cardiac markers and magnetic resonance imaging (MRI) findings, and cytologic analysis of the pericardial fluid did not reveal malignant cells, indicating an isolated immunologic etiology for the pericardial effusion. Following successful management, including oxygen support and a prednisone taper, chemotherapy without immunotherapy was resumed after a one-week delay. This rare case underscores the significance of promptly utilizing multimodality imaging with timely cardiology intervention, a prompt pericardial fluid analysis in diagnosing cardiac irAEs, and management leading to improved patient outcomes.

## Introduction

Immune checkpoint inhibitors (ICIs) have dramatically altered the landscape in cancer therapeutics. ICIs inhibit negative signaling pathways of immunomodulatory cells, therefore restoring immune surveillance of tumor cells. Atezolizumab is an ICI that inhibits the binding of programmed death-ligand 1 (PD-L1) to its inhibitory receptors, programmed cell death-receptor 1 (PD-1), and B7-1 expressed on T cells, consequently augmenting cytotoxic T-cell activity to elicit an anti-tumoral response. Its approval in 2019 in combination with etoposide and carboplatin marked a significant advancement in the first-line treatment of extensive-stage small-cell lung cancer [1]. However, the consequence of reactivating the immune system with such therapies can lead to immune-related adverse events (irAEs), which commonly involve the skin or gastrointestinal tract and less frequently, affect the cardiovascular system. A retrospective review of immune checkpoint inhibitor therapy and resulting pericardial effusion revealed that out of 3,966 cases, 15 required pericardiocentesis, with one attributed to atezolizumab [2]. Here, we present the case of a 67-year-old male recently diagnosed with stage IV metastatic small-cell lung cancer, who underwent treatment with carboplatin, etoposide, and atezolizumab, subsequently developing pericardial tamponade.

## Case presentation

**Figure 1:**
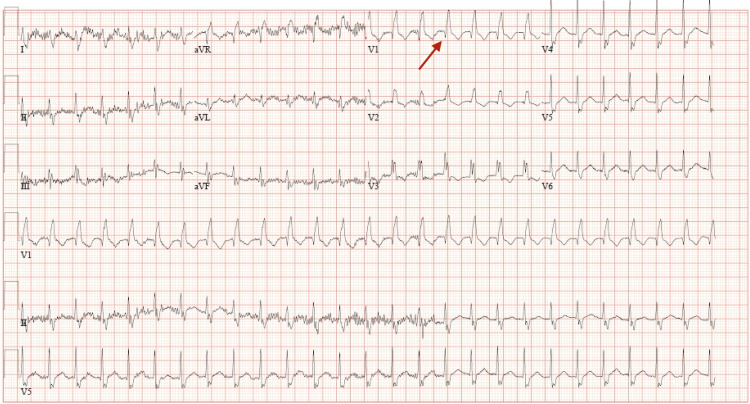
12-lead EKG at admit, showing afib with a rapid ventricular response at 158 beats per minute and RBBB with repolarization changes (arrow).

In early 2024, a 67-year-old male with a complex medical history, including a prior deep vein thrombosis managed with apixaban, atrial flutter treated with direct current cardioversion and ablation, and a significant smoking history of three-quarter packs per day for over 40 years, presented to the emergency department from his cancer infusion center due to hypoxia. He had recently been diagnosed with stage IV small-cell lung cancer with metastasis to the thoracic spine and was in the midst of carboplatin, etoposide, and atezolizumab therapy, having started the second cycle three weeks prior. However, acute hypoxic respiratory failure prevented him from completing the planned third cycle. On C3d3 of treatment, he presented with encephalopathy, hypotension, and hypoxia, prompting hospitalization. On admission, laboratory results revealed a leukocytosis of 13.2 x10^9/L, an elevated level of B-type natriuretic peptide (BNP) at 893 pg/mL, and a thyroid-stimulating hormone level within normal limits at 4.84 mIU/L. Creatinine levels were within normal limits at 0.7 mg/dL. Troponin-I levels were initially normal at <0.03 ng/mL and at 0.011 ng/mL on repeat draw, suggesting minimal to no cardiac muscle injury. The respiratory PCR panel yielded negative results, indicating the absence of respiratory viral pathogens. The initial electrocardiogram (EKG) demonstrated atrial fibrillation (Afib) with a rapid ventricular response at 158 beats per minute and right bundle branch block (RBBB) with repolarization changes (Figure 1), with atrial fibrillation being a new finding while RBBB remained unchanged from a previous EKG over a year prior.

**Figure 2:**
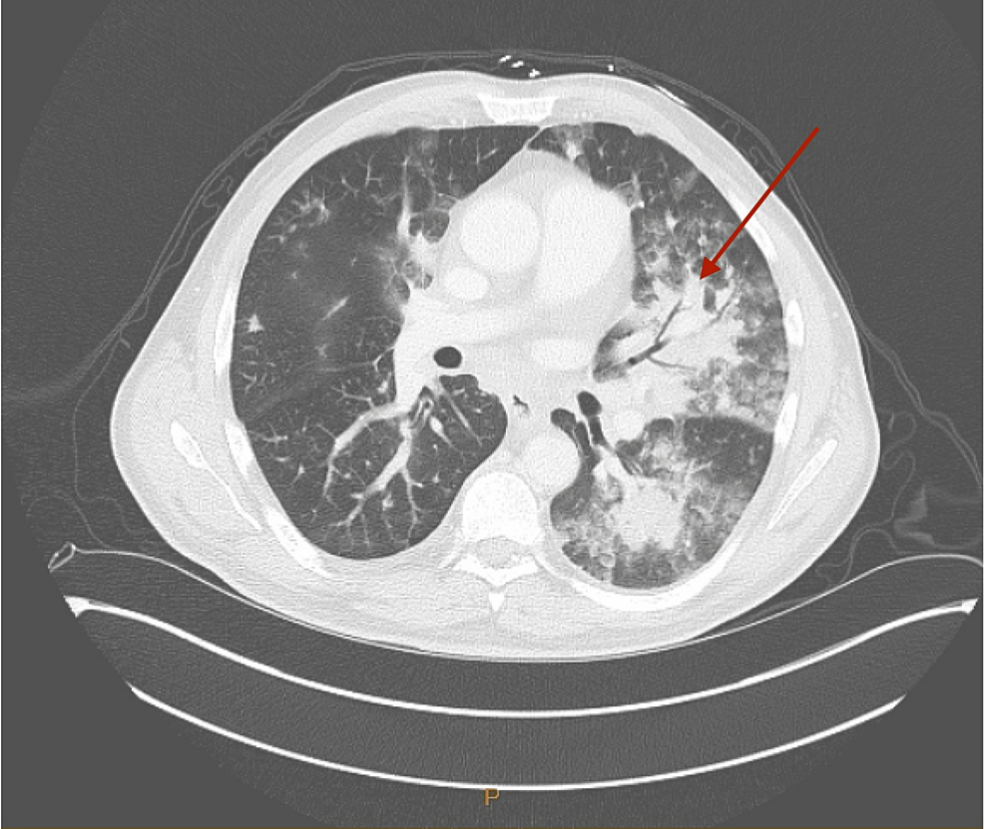
CT chest with contrast showing extensive left greater than right nodular airspace opacities and air bronchogram (arrow) consistent with multifocal pneumonia

The patient was admitted to the hospital for acute hypoxemic respiratory failure attributed to multifocal pneumonia observed on computed tomography (CT) chest with contrast (Figure 2).

**Figure 3:**
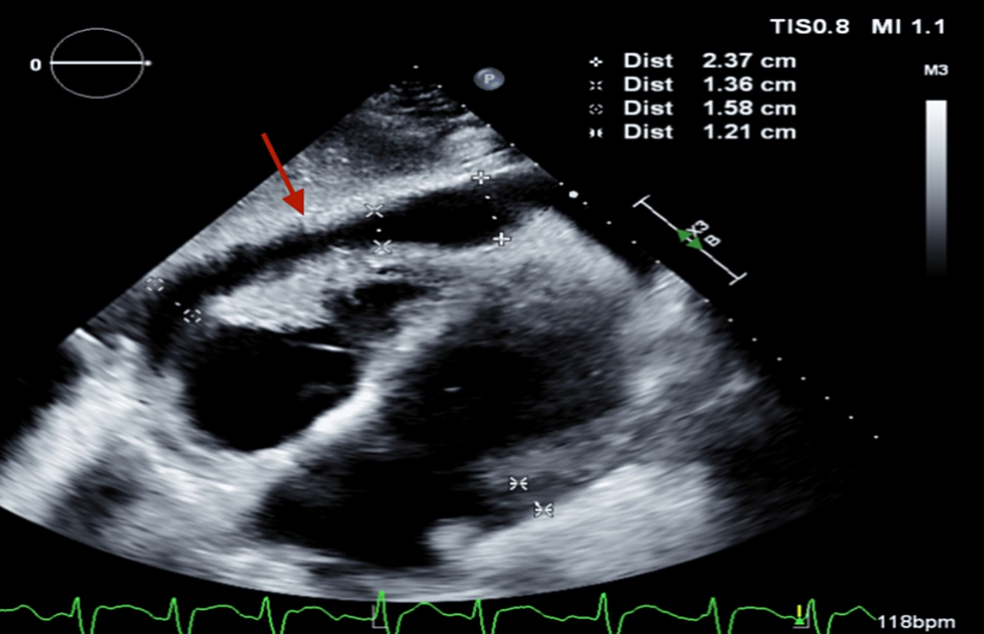
Subcostal view showing pericardial effusion (arrow)

**Figure 4:**
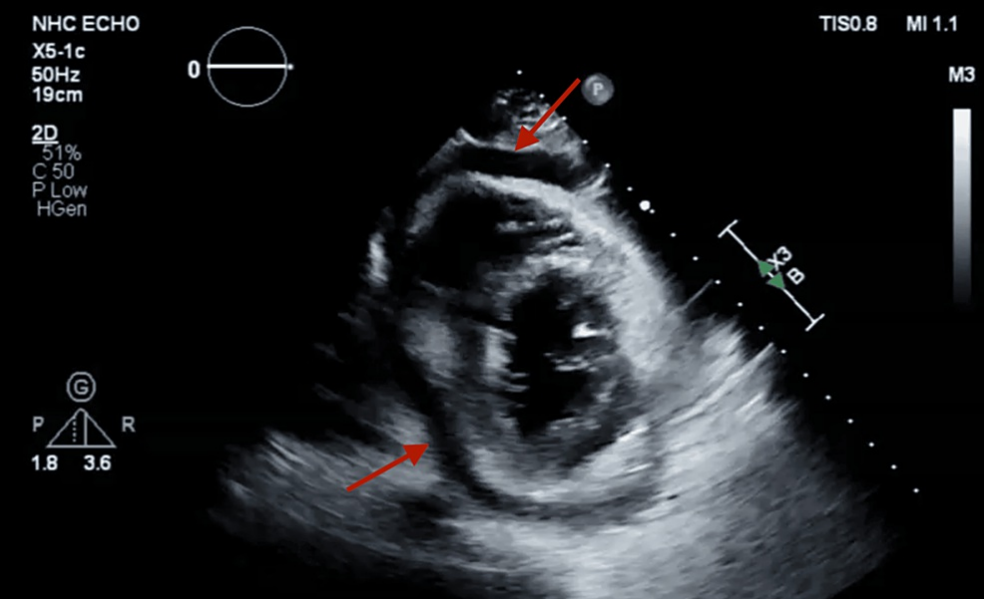
Short axis view showing pericardial effusion (arrow)

His initial transthoracic echocardiogram (TTE) showed mild to moderate circumferential pericardial effusion (1.3 cm) without evidence of tamponade. Throughout his hospitalization, his oxygen requirements increased, prompting repeat imaging. A limited TTE revealed significant findings, including mild-moderate pulmonary hypertension and a moderate-large circumferential pericardial effusion (Figures 3, 4) that had increased from previous assessments. There was no collapse of the right ventricle/right atrium, but the inferior vena cava was dilated with reduced respiratory variation. Significant transvalvular velocities were observed across the mitral valve and tricuspid valve, consistent with tamponade physiology.

**Figure 5:**
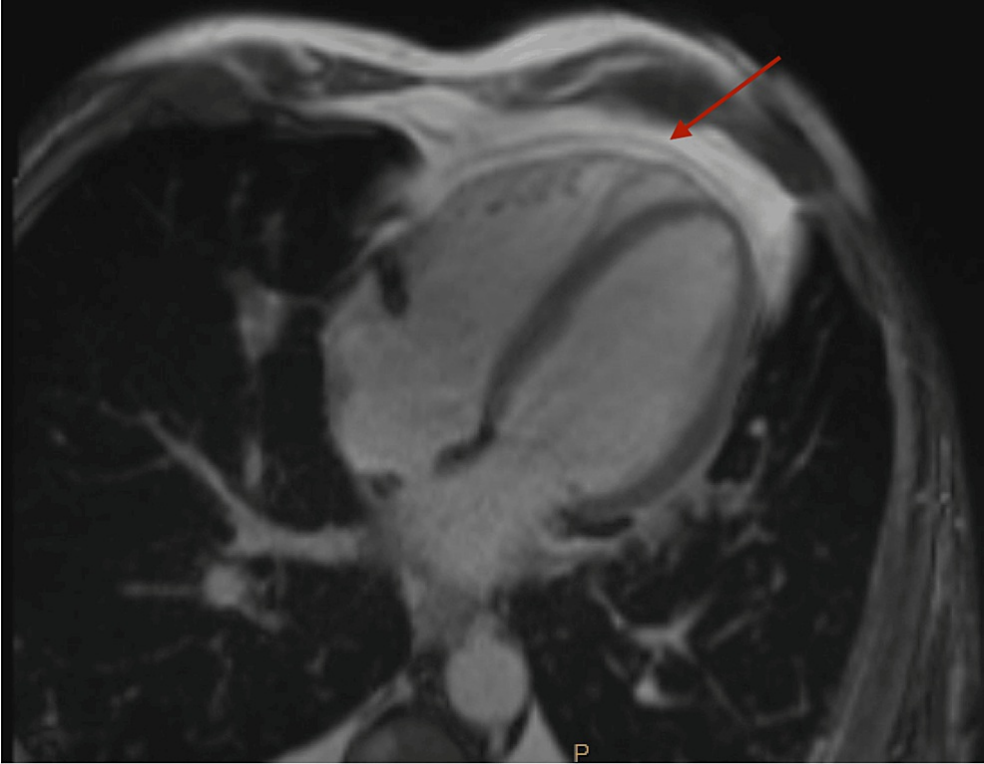
Cardiac MRI with contrast showing a trace pericardial effusion (arrow)

Pericardiocentesis was performed using ultrasound guidance via a subxiphoid approach, and a drain was placed. Pericardial fluid analysis revealed yellow, turbid fluid with a glucose level of 102 mg/dL, lactate dehydrogenase (LDH) level of 463 U/L, and protein level of 2.9 g/dL. Approximately 18,000 nucleated cells were observed, with negative gram stain, anaerobic culture, and acid-fast bacilli stain results. Cytology showed no malignancy but marked acute inflammation, with numerous neutrophils, leukocytes, histiocytes, and rare mesothelial cells noted. Cardiac magnetic resonance imaging (MRI) with contrast revealed a trace pericardial effusion. There were no abnormalities noted in the size and function of the left ventricle, indicating normal parameters. Additionally, there were no signs of delayed enhancement involving the left ventricular myocardium, suggestive of prior infarction, scar tissue, or infiltrative cardiomyopathy (Figure 5). The right ventricle appeared normal in size and function, with no observed wall motion abnormalities. Consequently, due to suspicion that his pericardial effusion was caused by immunotherapy, colchicine, and methylprednisolone at a dosage of 1 mg/kg/day were administered following established guidelines [3]. The pericardial drain was removed two days after the pericardiocentesis procedure was conducted.

The patient's hospital course was complicated by hypoxic respiratory failure, multifocal pneumonia, and pericardial effusion. Nonetheless, he responded well to the aforementioned interventions and was successfully weaned off oxygen support within days of starting high-dose steroids. He was discharged with a prednisone taper regimen and scheduled for close follow-up. Subsequently, after one month, he was able to resume chemotherapy without encountering additional complications.

## Discussion

Immune checkpoint inhibitors transformed cancer treatment through the enhancement of the natural immune response by binding to corresponding receptors on both tumor and T-cells, thus preventing inhibitory signaling and overcoming immune senescence. A consequence of reactivating immune surveillance is a myriad of irAEs, including cardiotoxicity. The pathophysiology of these adverse events is poorly understood, but one proposed mechanism suggests cross-reactivity related to molecular mimicry [4]. Examples of cardiotoxicity include cardiac arrest, heart failure, myocarditis, and pericardial disease. Although pericardial disease is especially rare, occurring at a rate of 0.36%, the associated mortality rate is high accounting for anywhere from 13-21% of fatalities [4-7]. The time course for ICI-related pericardial disease is usually within 30 days of treatment [8], presenting as dyspnea, chest pain, and hemodynamic instability [8,9], with dyspnea being the most common symptom [10]. Expected findings on imaging include EKG changes [8], fibrosis and/or pericardial inflammation on cardiovascular magnetic resonance (CMR), and pericardial effusion or thickening on CT [11]. The pericardial fluid analysis will show plasma cells and lymphocytes and can also include fibrinous exudate or hemorrhage but will not exhibit microorganisms or malignant cells [11-13]. A normal troponin can be used to rule out myocarditis, which rarely happens concurrently [14].

Typical side effects associated with atezolizumab include fatigue, cough, nausea and vomiting, dyspnea, alopecia, decreased appetite, constipation or diarrhea, rash, and headache, with rare manifestations that can potentially lead to therapy delays, including vasculitis, myocarditis, impaired ventricular function with heart failure, arrhythmias, and pericarditis [15]. The patient presented in this case was receiving atezolizumab in combination with carboplatin and etoposide as first-line therapy for extensive small lung cancer and had recent scans showing treatment response. His initial EKG revealed Afib with intermittent rapid ventricular response (RVR), and he was subsequently found to have pericardial effusion with tamponade physiology. The initial arrhythmia converted to sinus rhythm with the resolution of his pericardial effusion with pericardiocentesis, amiodarone, and initiation of steroids. The presence of inflammatory cells without malignant cells on pericardial fluid cytology distinguished the presentation from disease progression and pseudo-progression. Additionally, an unremarkable troponin-I level at 0.011 (normal range 0.00 - 0.028) and normal left ventricular systolic function without delayed enhancement of the LV myocardium on cardiac MRI suggests pericardial disease without myocarditis. Nonetheless, BNP could serve as a noninflammatory cardiac marker for myocarditis in a normal troponin-I cardiac marker.

A review of the literature on cardiotoxic irAEs did not reveal any other instances of pericardial effusions following the administration of atezolizumab that were not attributed to pseudo-progression. Pseudo-progression is characterized by a transient increase in tumor size followed by either regression or the appearance of new lesions. Unlike pseudo-progression, which typically resolves spontaneously, irAEs necessitate the discontinuation of ICI therapy, along with corticosteroid treatment for their immunosuppressive effects [16]. The presence of inflammatory cells without malignant cells on pericardial fluid cytology, coupled with the rapid recovery following temporary cessation of ICI therapy and a course of corticosteroids, distinguished this presentation from pseudo-progression and disease progression.

Guidelines recommend initiating high-dose systemic corticosteroid therapy when cardiac irAE is suspected [3]. In severe cases or if there is an inadequate response to corticosteroids, additional immunosuppressive agents, such as infliximab, mycophenolate mofetil, immunoglobulin, plasma exchange, tocilizumab, abatacept, cytotoxic T-lymphocyte-associated protein 4 agonists, or anti-thymocyte globulin, should be considered [3].

**Figure 6:**
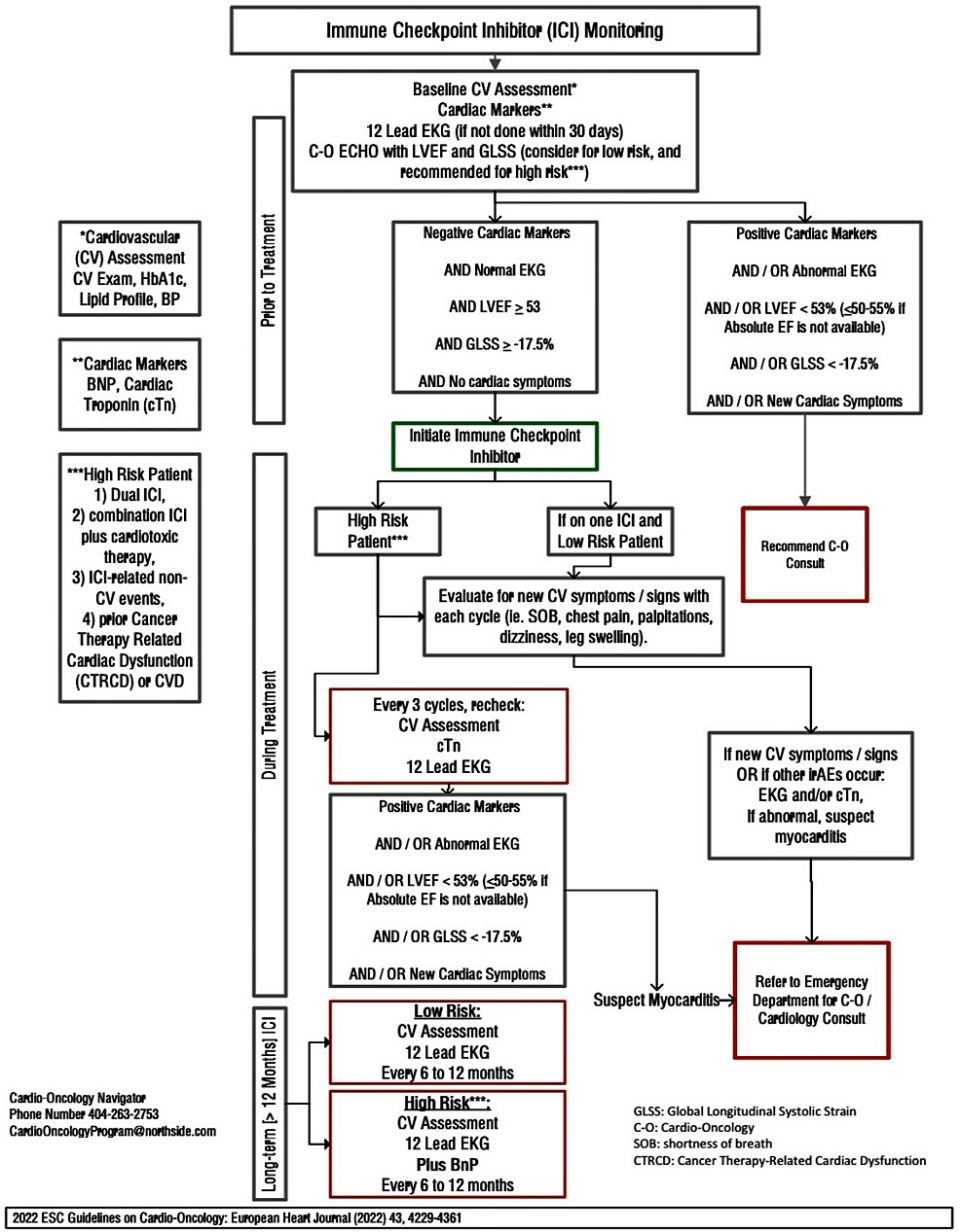
Northside Hospital Cardio-Oncology Program Risk Assessment algorithm for patients on immune checkpoint inhibitors CV: cardiovascular, HbA1c: hemoglobin A1c, cTn: cardiac troponin, CTRCD: cancer therapy-related cardiac dysfunction, SOB: shortness of breath, C-O: cardio-oncology, GLSS: global longitudinal systolic strain, CVD: cardiovascular disease, BnP: brain natriuretic peptide, LVEF: left ventricular ejection fraction, EKG: electrocardiogram, irAE: immune-related adverse events, ICI: immune checkpoint inhibitor This figure is the original work of the authors.

While pericardial toxicity associated with atezolizumab and other checkpoint inhibitors is rare, it poses a significant mortality risk. This underscores the critical importance of comprehensive cardiac monitoring and heightened awareness of potential cardiotoxicities, even before the initiation of treatment. To address this concern, the Northside Hospital Cardio-Oncology Program has implemented a Risk Assessment Algorithm for Patients on Immune Checkpoint Inhibitors, guided by recommendations from the European Society of Cardiology (ESC). This algorithm aims to mitigate cardiovascular risk factors and reduce the incidence of cardiovascular disease in cancer patients (Figure 6).

## Conclusions

In conclusion, this case report emphasizes the uncommon yet critical complication of cardiotoxic irAEs associated with ICI therapy, particularly atezolizumab, in the management of small-cell lung cancer. Despite its rarity, pericardial toxicity poses a significant mortality risk, underscoring the importance of comprehensive cardiac monitoring and heightened awareness of potential cardiotoxicities in patients receiving atezolizumab and other checkpoint inhibitors. Further investigations into the underlying mechanisms of cardiotoxic irAEs are imperative to mitigate their incidence and improve patient outcomes. Early diagnosis is paramount, especially considering that ICIs represent the frontline treatment for cancer, the widespread use of ICIs in both early and advanced-stage cancers, as single agents, and as part of combination chemotherapy. A multidisciplinary collaborative approach and the utilization of multimodality imaging are essential for the effective management of these patients.
